# Reproductive performance and productivity of local and Dorper x local crossbred ewes under community-based management system, Ethiopia

**DOI:** 10.1016/j.heliyon.2023.e19906

**Published:** 2023-09-06

**Authors:** Ayele Abebe, Gebreyohannes Berhane, Tesfaye Getachew, Solomon Gizaw, Aynalem Haile

**Affiliations:** a Debre-Birhan Agricultural Research, Amhara Agricultural Research Institute (ARARI), Debre-Birhan, Ethiopia; b Department of Animal Production Studies, College of Veterinary Medicine and Agriculture, Addis Ababa University, Ethiopia; c International Center for Agricultural Research in the Dry Areas (ICARDA), Addis Ababa, Ethiopia; d International Livestock Research Institute (ILRI), Addis Ababa, Ethiopia

**Keywords:** Age at first lambing, Annual reproductive rate, Crossbreeding, Dorper sheep, Litter size, Reproduction

## Abstract

This study evaluated the reproductive and productivity of local and Dorper crossbred ewes in a community-based management system. We analyzed data collected from 2013 to 2021, taking into account different factors such as dam-breed, location, type of birth, season, and year of lambing. Lambing was observed all year-round, but the majority (35%) occurred in September, October, and December. This suggests that pasture availability, which is influenced by climatic-factors, may play a role in the seasonality of lambing. Litter-size at birth and weaning did not show any significant difference. Age at first lambing varied between breeds (P < 0.001), while lambing-interval and annual reproductive rates were unaffected by dam-breed (P > 0.05). The annual number of lambing per year significantly varied based on location and lambing seasons (P < 0.001), with a higher frequency during the major rainy-season compared to the dry-season (1.58*vs*1.42), highlighting the influence of feed availability. Productivity indices of ewes were calculated. Location and season of lambing had a significant impact on annual ewe productivity, while the ewe genotype showed no significant influence on productivity indices, except for the weight of lambs produced per kilogram of metabolic weight (0.84*vs*0.72 lambs per kg ewe and year; P < 0.01: 2.02*vs*1.77 kg lamb per kg^0.75^ ewe and year), where local ewes outperformed Dorper crossbred ewes. The difference in annual-productivity indices between local and Dorper crossbred ewes was more evident when considering the postpartum weight, as the ewes exhibited higher postpartum weights. However, both ewe genotypes produced comparable lamb weights per year (20.91*vs*20.16 kg lamb weaned per ewe and year for local and Dorper crossbred ewes, respectively). In summary, under low-input conditions, Dorper crossbred ewes demonstrated comparable reproductive performances and productivity traits to local ewes. Nevertheless, breed and environmental factors identified in this study should be taken into account to enhance sheep productivity in both local and Dorper crossbred ewes.

## 1 Introduction

Sheep are an important part of Ethiopia's livestock production, distributed across various agro-ecologies in the country. According to Ref. [1], Ethiopia has 42.9 million heads of sheep. They play a significant role in uplifting rural communities and contribute to the socio-economic well-being of communities that rear them [2,3]. Sheep provide a source of protein and income for rural households, which helps to improve food security and reduce poverty. They are also a crucial part of traditional culture in many parts of Ethiopia and play a role in social ceremonies and events. The sale of sheep can provide income for households to pay for education and healthcare, which can help to improve the standard of living for families. Sheep farming also provides employment opportunities for many people, particularly in rural areas where jobs can be scarce. The demand for sheep and sheep products, both domestically and internationally, creates market opportunities for farmers and traders. According to Ref. [4], economic opportunities exist for small ruminant producers to supply animals to both the export and domestic markets. For instance, the [5] reported that the export of live sheep and goats generated over 74 million USD in revenue in the 2019/20 fiscal year. On the other hand, Sheep alone contributes 21% of the country's total ruminant livestock meat output, with the annual national mutton production estimated to be at 77 thousand metric tons [6]. These opportunities require overcoming many barriers to increased productivity, including nutrition, health, reproduction/genetics, marketing, and management.

In Ethiopia there is no specialized sheep breed maintained for specific use only; sheep in Ethiopia are mainly kept for fulfilling multiple roles, such as cash income, meat, milk, skin, wool, manure, security, gifts, and religious rituals [[7], [8], [9]]. Due to rapid urbanization, increased income levels, and population growth, the demand for and the price of sheep and goat meat has experienced a significant surge. For instance, the consumption of red meat has been growing at an annual rate of 5–6% in developing countries [10]. It is crucial to address this increasing demand for such products by promptly providing suitable alternatives making it a pressing and timely issue.

Despite their adaptability, sheep productivity in Ethiopia is low due to no targeted improvements for specialized purposes, resulting in dismal productivity levels of local sheep [11]. For instance, the average meat production per sheep in a given population is estimated to be between 3 and 3.5 kg per year [7]. To address this issue, crossbreeding using exotic sheep breeds has been suggested as a rapid method of improving breeds, and it has been successfully implemented in tropical regions to take advantage of breed complementarity [12]. In response to the performance gap observed in local sheep, the Ethiopia Sheep and Goat Productivity Improvement Program (ESGPIP) initiated a national Dorper crossbreeding program in Ethiopia. Dorper sheep are known for their rapid growth and excellent conformation, making them well-suited for meat production. The introduction of Dorper sheep genetics through crossbreeding aims to improve the productivity of the local sheep population in Ethiopia. Accordingly, ongoing crossbreeding initiatives are being implemented across multiple locations, namely *Basona Werana*, *Efratana Gidm*, *Kewet*, *Merhabete*, and *Kobo*, to enhance sheep productivity. These initiatives involve the introduction of exotic Dorper sheep to facilitate the improvement of local sheep breeds. In his review work [13], emphasized that sheep productivity, as it is related to food production, is influenced by multiple factors including reproductive efficiency. He highlighted that high reproductive output is a fundamental requirement for an efficient sheep production system. The significant contribution of reproduction to overall sheep productivity underscores the importance of genetic composition, as well as factors such as feed, health, and management practices.

Evaluating the performance of crossbred ewes at different stages of the crossbreeding work becomes crucial to enhance the productivity and profitability of sheep farms managed under community-based management systems. This study aims to evaluate the reproductive performances and productivity of local and Dorper crossbred ewes managed under farmers' conditions, which will provide insights into optimizing the reproductive efficiency of sheep and positively impact the overall productivity and profitability of sheep farms.

## 2 Materials and methods

### 2.1 Study sites, flock management, and mating system

**Fig. 1:**
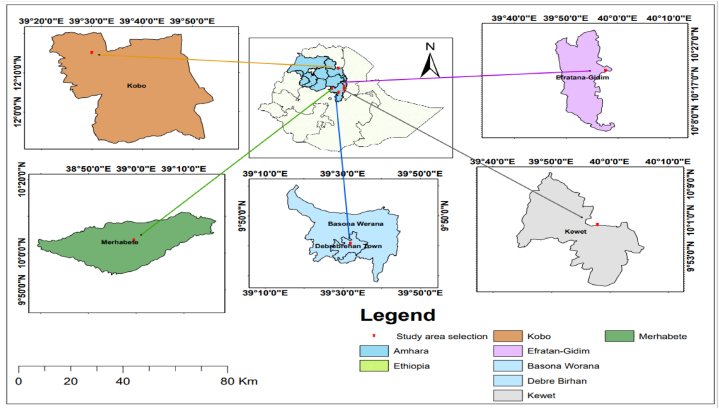
Map of the study areas.

This study was conducted on farmers' flocks in the Basona-Worana, Kewet, Efratana-Gidim, Merhabete, and Kobo areas, organized by the Debre-Birhan and Sirinka Agricultural Research Centers (Fig. 1).

The Basona-Werana (Milky) site has a crop-livestock production system, with natural pasture grazing serving as the primary feed resource accounting for 75% of the livestock's feed resources [14]. However, the availability of grazing land has been steadily decreasing. As a result, there has been an increased reliance on crop residues to meet the nutritional needs of livestock. Additionally, sheep in the area rely on wheat and barley stubble grazing as an additional feed resource. Despite the presence of industrial by-products such as wheat bran and brewers' spent grain, which are available in the areas, sheep are not currently being supplemented with these resources, instead, the leftover feed from cattle is being offered to them. This site itself is situated at an altitude of 2827 m. a.s.l., with coordinates 9° 40′ 35.97’’. The average temperature experienced is 13.5 ^O^c, and the area receives an average annual rainfall of 758 mm.

The Kewet site has a Sorghum producing farming system, with native pasture grazing serving as the primary feed resource for sheep [4]. Sheep also utilize grazing on Sorghum stubble as an additional source of feed. During the dry period, when grazing options are limited, sheep are supplemented with Sorghum Stover and Mung bean haulm to ensure their nutritional requirements are met. The site is located at an altitude of 1285 m. a.s.l with coordinates 10^0^ 0′ 0.12″ in the North and 39° 53′ 52.37″ in the east. The average temperature in this area is approximately 23.19 ^O^c and receives an annual rainfall of 1062.4 mm.

The Efratana-Gidim site is situated within a thriving farming system that cultivates major crops such as Sorghum, Teff, and Mung bean. Sheep mainly graze on natural pastures, with Sorghum stubble serving as an alternative feed resource. During the dry season, sheep are provided with Sorghum Stover and Mung bean haulm. The site is located at an altitude of 1448 m. a.s.l, with coordinates of approximately 10° 20′ 22.5″ north and 39° 57′ 38.28″ in the east. The region has an average temperature of 21.3 ^O^c and receives an annual rainfall of 1048 mm [15].

Merhabete/Alemketema is located in the Northern region at coordinates 10° 3′ 25.39″ in the north, and 38° 59′ 34.58″ in the east, with an altitude of 2225 m. a.s.l. and an average rainfall of 934 mm. The area has a mean temperature of 19.3 ^O^c and follows a crop-livestock mixed farming approach. Finger millet, Teff, and Maze are the primary crops in the midland area [16], while Sorghum, Mung bean, and Teff dominate in the lowland part [17]. Land allocation for grazing pasture in this study site is minimal, and sheep in the lowland parts graze on Sorghum and Teff stubble, which serves as their primary source of feed.

The Kobo (Raya-Kobo) site can be found at coordinates 12° 14′ 60.00″ in the North, and 39° 29′ 59.99″ in the East. The site sits at an altitude of 1470 m. a.s.l. and an average temperature of 23.1° Celsius. The area receives 630 mm of rainfall and is well-known for growing crops like Sorghum, Mung bean, and Teff. Livestock in the area relies on natural pastures and stubble grazing from Sorghum and Mung bean haulms for dry season feed sources.

This study focuses on several local ewe breeds: Menz in Basona-Werana, Afar in Kewet and Efratana-Gidim, local Merhabete in Merhabete, and Tumelie in Kobo areas. The crossbred ewes produced in this study had a blood level of Dorper ranging from 25% to 50%. In 2013, a community-Based (CBBP) Dorper crossbreeding program was initiated, involving volunteer farmers in the respective study areas. The number of households participating in the study was as follows: Basona-Werana (48), Merhabete (46), Efratana-Gidim (40), Kewet (42), and Kobo (45). On average, these households held 15, 9, 7, 8, and 11 sheep, respectively.

The crossbred ewes in the study have a blood level of Dorper ranging from 25% to 50%. In 2013, a community-based Dorper crossbreeding program was introduced, involving volunteer farmers in the respective study areas. The farmers received hands-on training covering various aspects of Dorper crossbreeding, lamb management, ram circulation, improved feeding systems, improved healthcare, and the concept of the community-based program. Once the farmers were aware of the utilization of Dorper rams, the distribution of Dorper rams from research centers took place. Farmers were grouped together based on proximity and their use of common grazing land and watering points. Each farmer group was allocated one sire to serve 20–25 breeding ewes, owned collectively by four to five households. The birth weight of the lambs and the postpartum body weight of the ewes were recorded within 24 h of lambing, along with lambing details. Rams were allowed to serve within the same breeding group for approximately one year before being transferred to another group within the same site to minimize inbreeding. The mating/crossbreeding system that followed was documented in a Dorper sheep strategy document [18,19]. The study flocks had already undergone deworming for internal parasites and spraying against external parasites prior to the study and every 6 months following the protocol from the research.

### 2.2 Traits considered and data collected

A team of researchers from the Debre-Birhan and Sirinka research centers collaborated to train a group of enumerators. The training focused on community-based crossbreeding practices, and the enumerators were responsible for collecting comprehensive data on reproductive performances from 2013 to 2021. The enumerators recorded the information in a master data-recording book prepared by the research team, with great attention to detail. To ensure the success of crossbreeding practices, diligent researchers made monthly visits to their respective flocks. This enabled them to address any challenges that arose and ensured the smooth execution of crossbreeding work. The collaborative approach between researchers, enumerators, and flock owners fostered an environment of continuous sheep crossbreeding practices.

Throughout the research project, various traits were evaluated to determine the flock's reproductive performance and productivity. These traits included litter size at birth and weaning, which shows the number of lambs successfully born and weaned per ewe. This data helped assess the breeding efficiency and overall reproductive success of the flock. Age at first parturition was also considered, which indicates the age at which ewes gave birth for the first time. This parameter served as an indicator of the flock's development and young ewes' ability to reproduce efficiently. The research also looked at the lambing interval, which indicates the duration between successive lambing events. This information helped assess the reproductive efficiency and breeding management practices employed within the flock. Annual reproductive rates were calculated to provide insight into the overall reproductive performance of the flock, including the percentage of ewes that successfully conceived and delivered lambs within a year. Additionally, two indexes were used to evaluate the productivity of individual ewes. The first index, Kilograms of lamb produced per ewe per year, quantified each ewe's productivity in terms of the weight of lambs produced within a year. It helped evaluate the genetic potential, feeding management, and overall performance of individual ewes. The second index, Kilograms of lambs produced per post-Partum weight of ewe per year, considered the weight of the ewe after lambing as a factor in assessing lamb productivity. It provided insights into the efficiency of utilizing the ewe's body condition and resources for lamb production. Finally, the third index, the Kilogram of lambs produced per metabolic post-partum weight of ewe per year, took into account the metabolic weight of the ewe after lambing, considering factors such as body condition and energy requirement. It provided a comprehensive assessment of lamb production efficiency based on the ewe's metabolic state.

The study analyzed a large dataset consisting of 3585 records for litter size at birth (LSB) and 2904 records for litter size at weaning (LSW). Additionally, 1493 records were used to evaluate the reproductive indices of ewes for Index-I, while Index-II and Index-III were based on 1181 records each. Index-I is a comprehensive measure of overall productivity that considers various factors such as lambing intervals and the weight of lambs at weaning (90 days of age). It is calculated by multiplying litter weight (kg) at 90 days of age by 365 days and dividing it by the subsequent lambing interval (days). Index-II is a refined version of Index-I that normalizes productivity against the live weight of the dam, allowing for a fair comparison across dams of different sizes. On the other hand, Index-III takes into account the metabolic weight of the dams, which is crucial in determining their specific energy requirements. It relates productivity to the metabolic (weight × ^0.73^) demands of the dams, providing insights into the efficiency of the breeding program [20].

• Index I: litter weight (kg) at 90 days of age × 365 days/subsequent lambing interval (days)
• Index II: index I/post-partum body weight of ewe.
• Index III: index I/metabolic post-partum body weight of ewe (kg)

### 2.3 Data analysis

For this study, we carefully managed and organized the collected data using Excel. To find valuable patterns and relationships within the data set, we conducted a rigorous analysis using General Linear Model procedures within the Statistical Analysis System [21]. The response variables we investigated included key metrics such as LSB, LSW, ARR, AFL, LI, and Indices I-III. We considered several fixed effects, such as the location of the study, the breed of the dam, the lambing season, and the year in which the data were collected. We also included the dam's parity (the number of times it has given birth) and the sex of the lamb as fixed effects. When the analysis of variance indicated significant differences, we used the Tukey-Kramer Test to compare the least squares means and determine any significant variations among the different levels of factors. Our thorough investigation of the data, with the consideration of these fixed effects and the appropriate statistical analysis, yielded meaningful findings for the study. To calculate the lambing distribution, we used frequency analysis. We employed the PROC GLM procedure [21] to analyze factors such as LSB, LSW, AFL, LI, ARR, and various indices. The dataset comprised 389 ewe records for AFL analysis and 298 records for both LI and ARR analysis.

The statistical model employed for reproductive performances traits was.

Model 1 Analysis of variance of Litter size at birth and weaning.

Yijklm = μ + Li + Dj + Sk + Yl + Pm + Gn + *ijklmn*, Where;

Yijklmn = the observation on Litter size at birth and weaning

μ = Overall Least squares mean.

Li = Fixed effect of i^th^ **Location** of the study Flock (i = 1–4: Baso; Kewet-EF; Kobo and Merhabete).

Dj = Fixed effects of j^th^ **dam** genotype (j = 2: Local; Dorper crossbred).

Sk = Fixed effects of kth lambing **season** (k = 3: Dry-period; Long-rainy; Short-rainy).

Yl = Fixed effects of lth lambing **year** (l = 9: 2013, 2014 … 2021).

Pm = Fixed effects of mth dam lambing **parity** (m = 5: 1, 2, 3, 4, ≥5).

Gn = Fixed effects of nth lamb **Gender** (G = 2: Male; Female).

*eijklmn* = effect of random error.

Model 2 Analysis of variance of Age at first lambing, Lambing interval, and Annual reproductive rate.

Yijklm = μ + Li + Dj + Sk + Yl + Pm + *ijklm*, Where;

Yijklm = the observation on Age at first lambing, Lambing interval, annual reproductive rate, and annual productivity indices of a ewe (Index-I, Index-II, and Index-III).

μ = Overall Least squares mean.

Li = Fixed effect of i^th^ **Location** of the study Flock (i = 1–4: Baso; Kewet-EF; Kobo and Merhabete).

Dj = Fixed effects of j^th^ **dam** genotype (j = 2: Local; Dorper crossbred).

Sk = Fixed effects of kth lambing **season** (k = 3: Dry-period; Long-rainy; Short-rainy).

Yl = Fixed effects of lth lambing **year** (l = 9: 2013, 20,114 … 2021).

Pm = Fixed effects of mth dam lambing **parity** (m = 5: 1, 2, 3, 4, ≥5).

*eijklm* = effect of random error.

## 3 Results and discussion

### 3.1 Reproductive performance

#### 3.1.1 Lambing pattern and lambs’ birth weight distribution

**Fig. 2:**
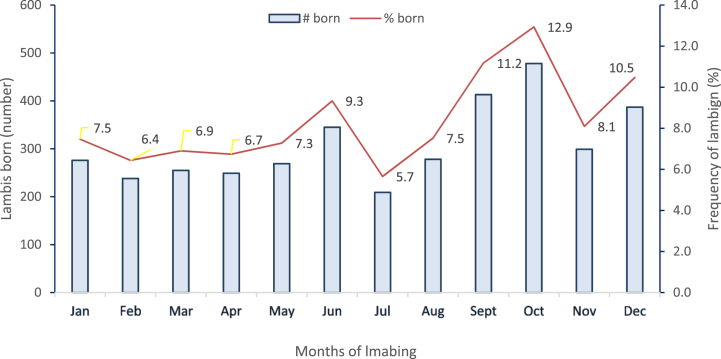
Monthly lambing frequency of local and Dorper crossbred lambs in the study areas (n = 3585).

It is important to understand the lambing pattern and distribution of lamb birth weight in sheep for assessing their reproductive efficiency and overall productivity. Sheep in temperate regions usually have a seasonal breeding pattern, while the presence of rams in tropical flocks often results in lambing occurring throughout the year [22,23]. Our study found that lambing was distributed across all months, with an average of 6%–13% of ewes lambing per month (Fig. 2). This suggests that ewes in our study were capable of coming into heat at any time of the year, regardless of their breed, whether local or Dorper crossbred. Although lambing occurred throughout the year, our study identified a peak lambing period in September, October, and December, accounting for 35% of total lambing. This is similar to a previous study conducted on Tamilnadu flocks, where the peak lambing season was reported to be in November followed by October and December [24]. However, lambing events were still present throughout the year. In contrast, different lambing peaks were reported for Karakul, Afrikander, and Blackhead Persian sheep and their various crosses, with the highest proportions occurring in April, May, and August (16%, 17%, and 19%, respectively) by Ref. [25]. Furthermore, higher proportions of lambs were reported to be born in January and February for the Washera and Farta sheep breeds [22]. Similarly, considerable lambing was found to occur in January, February, May, and June in studies on Djallonke sheep [26].

**Fig. 3:**
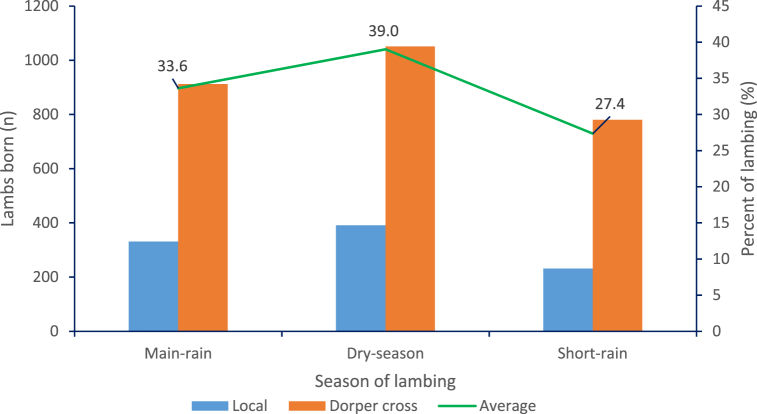
Seasonal distribution of lambing for local and Dorper crossbred lambs.

The study showed that both local and Dorper crossbred ewes had similar seasonal lambing patterns, with 39% of the ewes giving birth during the Dry season while 33% gave birth during the main rainy season. This suggests that a larger number of ewes in both breeds entered estrus during the rainy season (Fig. 3). These findings are consistent with previous studies conducted on Algerian sheep and ewe breeds raised in challenging climatic conditions in Southwestern Algeria [27]. Likewise [28], concluded that ewe breeds raised in challenging climatic conditions in Southwestern Algeria exhibited relatively non-seasonality, with only a slight decrease in productive activity. It was noted that ewes in tropical and sub-tropical environments are either completely seasonal or intermittently polyestrous [29]. The quality and availability of feed can influence breeding activity. Previous studies have suggested that seasonal variation in feed supply can influence the lack of seasonality in estrus behavior among tropical sheep breeds [30]. Despite the Dorper breed being managed under improved systems, the study found that the crossbred ewes still maintained good reproductive performance. The study provides valuable insights into the lambing pattern and distribution of lambing in sheep. It highlights the absence of a clear seasonality in lambing for both local and Dorper crossbred ewes. The identification of peak lambing periods and the influence of seasonal factors on reproductive activity can inform management practices aimed at enhancing productivity.

#### 3.1.2 Litter size

##### 3.1.2.1 Litter size at birth (LSB)

The litter size at birth (LSB) is a crucial factor in measuring the reproductive performance of sheep. Our study found an overall birth litter size of 1.09 ± 0.03 (3585) (Table 1). We observed that the birth litter size of local sheep and Dorper crossbred sheep was 1.06 ± 0.03 and 1.05 ± 0.03, respectively. These results are consistent with previous research conducted by Ref. [31], which reported litter sizes ranging from 1.05 to 1.15 in tropical breeds. Similarly [22], found litter sizes of 1.03 and 1.05 for local Washera sheep under the station and on-farm management, respectively [32]. also reported litter sizes of 1.03 for local ewes and 1.04 for Awassi crossbred ewes, further supporting our findings. When comparing our results to other studies, we found that the average litter size of local and Dorper crossbred sheep in our study was lower than the value of 1.28 reported by Ref. [33] for Dorper sheep. However, it was higher than the 1.02 litter size reported by Ref. [34] for Dorper sheep under extremely unfavorable conditions. It's important to note that litter size can be influenced by various factors, such as genetic factors, environmental conditions, and management practices. Our study found that LSB was significantly influenced by location, ewe parity, lamb sex, the season of lambing (P < 0.001), and the lambing year of ewes (P < 0.05). These findings differ from the results reported by Ref. [35] for Garole sheep raised under semi-arid conditions, where the season of lambing did not significantly affect LSB. However, in line with [23], we also found a significant effect of ewe parity on LSB.

**Table 1:** Least squares means (±SE) of the effects of location, Dam genotype, Lambing season, Lambing year, Parity, and Lamb sex on litter size at birth and weaning for Local and Dorper crossbred sheep.

Source of variation	LSB		LSW	
	N	LSM±SE	N	LSM + SE
**Overall**	**3585**	**1.10** ± **0.03**	**2904**	0.95 + 0.30
**CV (%)**	**3585**	**26.48**	**2904**	32.03
**Location**		***		***
*Basona-Werana*	1709	1.10^a^±0.03	1459	1.00^a^+0.04
*Kewet-EF*	941	1.03^b^ ± 0.03	584	0.90^b^ + 0.04
*Kobo*	460	0.99^b^ ± 0.03	435	0.95^b^ + 0.04
*Merhabete*	475	1.09^a^+0.04	426	0.92^b^ + 0.04
**Dam Genotype**		ns		ns
*Local*	3242	1.06 ± 0.03	2611	0.94 + 0.03
*Dorper crossbred*	343	1.05 ± 0.03	293	0.94 + 0.04
**Lambing Season**		***		***
*Dry season*	1396	1.07^a^±0.03	1149	0.92^b^ + 0.03
*Major rainy season*	1235	1.03^b^ + 0.03	998	0.93^b^ + 0.04
*Miner rainy season*	964	1.06^a^±0.03	757	0.98^a^+0.04
**Lambing Year**		**		**
*2013*	69	1.05^a^±0.04	67	0.99^a^+0.04
*2014*	324	1.09^a^±0.02	187	0.94^b^ + a.03
*2015*	580	1.12^a^±0.02	408	0.90^b^ + 0.02
*2016*	1068	1.11^a^±0.01	957	0.90^b^ + 0.02
*2017*	979	1.08^a^±0.01	828	0.92^b^ + 0.02
*2018*	248	1.04^b^ ± 0.02	141	0.77^c^+0.03
*2019*	20	1.05^a^±0.07	19	1.05^a^+0.07
*2020*	220	1.10^a^±0.02	220	1.05^a^+0.03
*2021*	77	0.99^b^ ± 0.03	77	0.97^a^+0.04
**Parity of the Dam**		**		ns
*1*^*St*^*Parity*	2270	1.02^b^ ± 0.03	1827	0.97 + 0.03
*2*nd *Parity*	793	1.06^a^±0.03	640	0.94 + 0.04
*3*rd *Parity*	307	1.03^b^ ± 0.04	264	0.95 + 0.04
*4*th *Parity*	129	1.08^a^±0.04	99	0.95 + 0.04
*5*th *& above parity*	86	1.08^a^±0.04	74	0.91 + 0.05
**Lamb Sex**		*		**ns**
*Male*	1805	1.07 ± 0.03	1430	0.94 + 0.03
*Female*	1780	1.04 ± 0.03	1474	0.95 + 0.03

LSB (Litter Size at Birth), LSW (Litter Size at Weaning), LSM (Least Squares means), SE (Standard errors).

There are several factors that can affect the size of a litter when sheep give birth. These include ovulation rate, uterine capacity, age at first lambing, parity, and the body condition of the ewe [36,37]. By improving the environment in which the sheep live, such as ensuring they have enough high-quality feed and using appropriate management techniques, it is possible to increase their productivity. Our study found that local and Dorper crossbred sheep have comparable litter sizes, which highlights the importance of considering factors such as location, ewe parity, lamb sex, season of lambing, and lambing year when assessing reproductive performance. To improve litter size and overall productivity in sheep breeding programs, further research and targeted management strategies should be implemented.

##### 3.1.2.2 Litter size at weaning (LSW)

The number of lambs in a litter at weaning (LSW) is an important factor in determining the success of reproduction and survival rates. In our research, the average litter size at weaning was found to be 0.95 ± 0.30 (Table 2). Interestingly, we discovered no significant differences in litter size at weaning between the local breed and the Dorper crossbred ewes (0.943 ± 0.03 and 0.944 ± 0.04; *P* > 0.05, respectively), indicating that introducing Dorper genetics did not have a major impact on the number of lambs weaned compared to the local breed. When compared to previous studies, our litter size at weaning values was slightly lower than those reported by Ref. [38] for Abera sheep under the CBBP in Ethiopia, which reported a LSW of 0.978. However, it is important to keep in mind that various factors, such as environmental conditions, management practices, and genetic variations, can contribute to variations in litter size at weaning across studies. On the other hand, our litter size at weaning values for both the local and Dorper crossbred ewes were higher than those reported by Ref. [39] for hair sheep under annual and accelerated lambing programs, where litter size at weaning ranged from 0.56 to 0.72 for the annual system mating and 0.46 to 0.94 for the accelerated system, calculated on a per lambed ewe basis.

**Table 2:** Least squares means (±SE) of the effects of location of the flock, Dam genotype, Lambing year aa d season on average birth and weaning litter size of Local and Dorper crossbred sheep.

Source of variation	AFL		LI		ARR	
	N	LSM *+* SE	N	LSM±SE	N	LSM + SE
**Overall**	389	15.51 + 0.43	298	9.36 ± 0.39	298	1.47 + 0.10
**CV (%)**	389	18.39	298	22.49	298	25.91
**Location of the Flock**		***		**		***
*Basona-Werana*	89	16.47^a^+0.31	128	10.57^a^±0.64	128	1.43^b^ + 0.05
*Kewet-EF*	57	15.44^abc^+0.38	74	9.29^a^±0.46	74	1.41^bc^+0.05
*Kobo*	191	15.78^ab^ + 0.22	75	9.87^a^±0.27	75	1.27^c^+0.05
*Merhabete*	52	14.10^c^+0.40	21	7.91^b^ ± 0.72	21	1.83^a^+0.09
**Dam Genotype**		***		ns		ns
*Local*	231	14.69 + 0.23	265	9.01 ± 0.16	265	1.55 + 0.03
*Dorper crossbred*	155	16.21 + 0.23	33	9.67 ± 0.39	33	1.42 + 0.07
**Lambing Season**		ns		ns		***
*Dry season*	136	15.29 + 0.26	101	9.56 ± 0.28	101	1.42^b^ + 0.05
*Major rainy season*	155	15.48 + 0.24	108	8.98 ± 0.28	108	1.58^a^+0.05
*Miner rainy season*	98	15.57 + 0.30	89	9.47 ± 0.28	89	1.44^b^ + 0.05

Our study found that birth year and location significantly impact litter size at weaning (LSW) (P < 0.001), indicating that temporal and spatial factors are crucial in determining LSW. The differences in LSW across various birth years and locations can be attributed to the varying environmental conditions, management practices, and other location-specific factors. However, our study did not find a significant effect of dam genotype, parity of the dam, and lambing seasons on LSW (P > 0.05), suggesting that their influence on LSW may be relatively limited in our specific context. Our findings provide valuable insights into litter size at weaning in both local and Dorper crossbred sheep. Interestingly, there were no significant differences in LSW between the two breeds, indicating that the introduction of Dorper genetics did not have a substantial impact on LSW. Our results emphasize the importance of considering birth year and location when assessing reproductive performance. Further investigation into the underlying causes and potential management strategies can help improve LSW and enhance overall productivity in sheep farming.

#### 3.1.3 Age at first lambing

The age at which sheep give birth for the first time, known as Age at First Lambing (AFL), is an important reproductive factor that reflects how efficient sheep production systems are. Our study found that the overall AFL was 15.51 months (Table 2). Interestingly, the AFL for local ewes was significantly lower at 14.69 months compared to Dorper crossbred ewes at 16.21 months. This suggests that differences in feed availability and management practices among locations may have played a role in this variation, as the significant effect of location on the AFL (P < 0.001) indicates.

We did not observe any significant effects of lambing year or season on the AFL (P > 0.05), indicating that these factors did not substantially influence the age at which the ewes gave birth to their first lamb in our population. Our findings align with previous reports from tropical regions by various authors [40,41], which also observed AFL values of 15–16 months. Additionally, our AFL values are similar to those obtained by Ref. [42] for the Bonga sheep breed under the CBBP (15.1 months), as well as the results reported by Ref. [22] for Washera sheep under on-station and on-farm management systems (15.7 and 15.2 months, respectively).

Our study found that indigenous local sheep have an average age at first lambing (AFL) of 14.69 months, which is slightly higher than what other authors have reported for local sheep breeds in different regions of Ethiopia. For example, Gumuz sheep have an AFL of 13.67 months; [43], Afar sheep have an AFL of 13.52 months [44], and Horro sheep have an AFL of 13.3 months [8]. However, Dorper crossbred ewes have a higher AFL value of 16.21 months compared to the reported values from the same authors. It's important to mention that a different study [45] found that indigenous Menz sheep have AFL values slightly higher at 15.67 months. On the other hand, a preliminary observation by Ref. [46] reported lower AFL values of 12.5 months for Dorper crossbred ewes under farmers' management in Southern parts of Ethiopia (Dorper x Local 50%) [47]. also reported AFL values ranging from 14.9 to 16.5 months for Menz, Corriedale Crossbred, and Awassi Crossbred ewes in Ethiopia. Improving AFL is crucial to enhance the lifetime productivity of ewes. Farmers can achieve this by selecting and breeding ewes at an earlier age (7–9 months) instead of waiting until they are 18 months old [48]. By focusing on early breeding, farmers can optimize the reproductive efficiency and lifetime performance of their sheep flocks. In conclusion, our study sheds light on the AFL of local and Dorper crossbred sheep. The significant impact of location on AFL highlights the importance of considering geographical factors and management practices. Further research and interventions aimed at optimizing AFL can contribute to improved productivity and sustainability in sheep production systems.

#### 3.1.4 Lambing interval

In the study we conducted, we examined the lambing interval (LI) of local and Dorper crossbred sheep. LI is an important factor in determining the reproductive effectiveness of a ewe flock. Our findings showed that the overall LI was 9.36 ± 2.11 months (Table 2). Local ewes had an LI of 9.01 ± 0.16 months, while Dorper crossbred ewes had an LI of 9.67 ± 0.39 months. This slight variation suggests that genetic factors may play a role in determining the time interval between successive lambing. Interestingly, we did not observe any significant effects of ewe genotypes and ewe lambing season on LI (P > 0.05). This implies that other factors may have a more substantial influence on the lambing interval in our study population. However, we did find that location had a significant effect on LI (P < 0.05), indicating that environmental factors, management practices, and local conditions may affect the duration between lambing. When comparing our LI results to previous studies, we found that the LI for local ewes in our study (9.01 months) was similar to the reported values of 9 months for local ewes by other researchers. Additionally, the LI value for Dorper crossbred ewes (9.67 months) in our study was slightly higher than the reported LI for Awassi crossbred ewes by Ref. [32]. Our LI results fell within the range of estimates (7.7–14.6 months) reported for various sheep populations in Africa [49], indicating that the lambing interval observed in our study is consistent with regional patterns.

After analyzing values over different seasons, we discovered slight variations. During the dry, major rainy, and minor rainy seasons, the lambing intervals were 9.56 ± 0.28, 8.98 ± 0.28, and 9.47 ± 0.28 months, respectively. Although these differences were not statistically significant, our findings suggest that seasonal variations may have some influence on the lambing interval. Our study offers valuable insights into the lambing interval of local and Dorper crossbred sheep in the study area. These findings emphasize the significance of considering genetic factors, environmental conditions, and management practices when striving to improve reproductive efficiency and lambing intervals in sheep flocks. Further research is necessary to explore additional factors that may impact the lambing interval, such as nutrition, health status, and hormonal regulation, in order to develop targeted strategies for enhancing reproductive performance in various sheep production systems.

• AFL (Age at first lambing, months), LI (Lambing interval, months)
• Lambing season: Dry-Period (October–January), Long-Rain (June–September), Short-Rain (February–May)
• ARR (Annual Reproductive rate), Index-I (annual litter weight at 90 days x number of lambing per ewe; Index-II (Index-I/Post-partum weight of dam in kg; Index-III (Index-I/metabolic post-partum weight of the dam)

### 3.2 Estimates of productivity

#### 3.2.1 Annual reproductive rate (ARR)

In the study, it was found that the average number of lambs per ewe per year was 1.47 (Table 2). During different seasons, the number of lambing per ewe per year varied by 1.42 in the dry season, 1.58 in the major rainy season, and 1.44 in the minor rainy season. When comparing the two genotypes, local ewes produced 1.55 lambs per ewe per year, while Dorper crossbred ewes produced 1.42 lambs per ewe per year. The location and lambing season had a significant effect (P < 0.001) on the ARR, while ewe genotype and lambing year did not have any significant effect (P > 0.05). This lack of effect could be due to variations in environmental factors and climatic conditions that can affect pasture availability. Interestingly, Dorper crossbred ewes had a similar number of lambs per ewe per year compared to local ewes. It is worth noting that the results of this study slightly exceeded the findings of [31], who reported 1.4 and 1.2 lambs per ewe per year for local and Awassi ewes, respectively.

The ARR values obtained for local and Dorper crossbred sheep (1.55 and 1.42, respectively) were lower than the reported number of lambing per ewe per year for Abera sheep (1.9) by Ref. [38]. However, the ARR for Dorper crossbred ewes in our study closely aligned with the findings of [25] for Karakul sheep in Botswana (1.42 vs 1.45). Additionally, our results (1.55 and 1.42) for local and Dorper crossbred ewes exceeded the values reported by Ref. [22] for Washera and Farta sheep (1.38, 1.29, respectively) under on-farm management. These differences in ARR could be attributed to variations in ewe genotypes and geographical locations. Improving the efficiency of meat production in sheep could potentially be achieved by focusing on strategies to enhance reproductive performance and increase the number of lambs marketed per ewe per year [39].

#### 3.2.2 Annual production indices

This study evaluated the productivity of ewes in terms of lamb rearing using three annual production indices: Index-I, which measured the kilogram of lambs reared to 90 days; Index-II, which measured the kilograms of lambs reared per kilogram of ewe postpartum weight; and Index-III, which measured the kilograms of lambs reared per kilogram of ewe metabolic weight. The study found that the overall productivity indices were: Index-I: 19.55 kg, Index-II: 0.76 kg, and Index-III: 1.82 kg (Table 3).

**Table 3:** Least squares means (±SE) of the effects of location of the flock, Dam genotype, Lambing season, Parity on subsequent productivity indices of Local and Dorper crossbred sheep.

Source of variation	Index-I		Index-II			Index-III	
	N	LSM + SE	N	LSM±SE	N		LSM + SE
Overall	1493	19.55 + 0.59	1181	0.76 ± 0.03	1181		1.82 + 0.07
**CV %**	1493	39.27	1181	40.65	1181		40.15
**Location of the flock**		***		***			***
*Basona-Werana*	699	18.99^c^+0.50	699	0.69^c^±0.03	699		1.68^c^+0.06
*Kewet-EF*	311	20.85^b^ + 0.57	311	0.79^b^ ± 0.03	311		1.90^b^ + 0.06
*Kobo*	312	19.00^c^+0.49					
*Merhabete*	171	23.29^a^+0.71	171	0.86^a^±0.03	171		2.10^a^+0.08
**Dam genotype**		ns		**			**
*Local*	1327	20.91 + 0.33	1111	0.84 ± 0.02	1111		2.02 + 0.04
*Dorper crossbred*	166	20.16 + 0.68	70	0.72 ± 0.04	70		1.77 + 0.09
**Lambing season**		**		***			***
*Dry season*	576	19.50^b^ + 0.47	461	0.73^b^ ± 0.03	461		1.77^b^ + 0.06
*Major rainy season*	529	21.16^a^+0.50	411	0.79^a^±0.03	411		1.93^a^+0.07
*Miner rainy season*	388	20.94^a^+0.54	309	0.82^a^±0.03	309		1.99^a^+0.07
**Parity**		*		ns			ns
*1*st	614	19.27^b^ + 0.41	509	0.73 ± 0.02	509		1.76 + 0.05
*2*nd	520	20.26^a^+0.43	424	0.75 ± 0.02	424		1.82 + 0.05
*3*rd	222	20.64^a^+0.59	162	0.78 ± 0.03	162		1.89 + 0.07
*4*th	81	21.04^a^+0.91	54	0.78 ± 0.05	54		1.89 + 0.11
*5*th *& above parity*	56	21.46^a^+1.08	32	0.86 ± 0.06	32		2.09 + 0.14

The body weight of lambs weaned was similar between the local and Dorper crossbred ewes, with weights of 20.91 kg and 20.16 kg, respectively. Although not statistically significant, the local ewes showed slightly better performance, which could be attributed to their slight advantages in LSB (litter size at birth) and LI (lambing interval). In another study conducted by Ref. [25] on Karakul sheep in Botswana, they obtained a lamb production rate of 27.7 kg per ewe per year, which significantly surpassed the results obtained in our study for both the local and Dorper crossbred ewes. Compared to previous studies on hair sheep [39], our study achieved higher kilogram values of lambs weaned per ewe per year. Therefore, our study demonstrates improved productivity levels. However, when examining the total weight of lambs at weaning per kilogram of metabolic weight and per kilogram of ewe weight, significant (P = 0.0024) differences were observed. The local ewes performed better than the Dorper crossbred ewes, with weight values of 0.84 kg versus 0.72 kg and 2.01 kg versus 1.76 kg, respectively. This is possible because the local ewes are smaller in size. Furthermore, the study found that the values of the indices increased with the advancing parity of the dam, which aligns with the patterns observed in Djallonke sheep as reported by Ref. [50]. The difference in annual productivity indices between the local and Dorper crossbred ewes was more pronounced when considering the postpartum body weights. Similarly, the reduced productivity of the Dorper crossbred ewes became more evident when productivity was expressed per kilogram of ewe weight, due to a slight difference in lambing interval. In summary, the results indicate that the local ewes exhibited slightly better productivity compared to the Dorper crossbred ewes, likely due to their smaller size and more favorable lambing interval. Our study achieved higher levels of lamb production per ewe per year compared to previous studies, although further improvements can be made by optimizing the management practices for Dorper crossbred ewes.

## 4 Conclusions and recommendations

In this study, it was discovered that both local and Dorper crossbred ewes are capable of lambing throughout the year. The distribution of lambing was found to range from 6% in July to 13% in October. While the majority of lambing occurred during the dry season (39%), there was no clear seasonality in lambing for either breed. It was noted that Dorper crossbred ewes performed well under a smallholder low-input production system, which is a great advantage of the breed. In contrast, other exotic breeds may not survive under such conditions. Environmental factors were found to have a significant impact on litter size and other productivity indices, but the ewe genotype did not. The results suggest that local ewes exhibited slightly higher productivity than the Dorper crossbred ewes, possibly due to their smaller size and more favorable lambing. However, it was noted that further improvements in management practices for Dorper crossbred ewes could enhance productivity.

## Authors’ contributions statement

Ayele Abebe: Conceived and designed the experiment; performed the experiment; acquisition of data; analyzed and interpreted the data; contributed materials; wrote the paper; critically revised the paper; final approval of the version to be published. Gebreyohannes Berhane and Aynalem Haile: Designed the experiment; analyzed and interpreted the data; contributed materials; guided the project; wrote the paper, and final approval of the version to be published. Solomon Gizaw and Tesfaye Getachew: Conceived and designed the project, performed the experiment, analyzed and interpreted the data; and wrote the paper; critically revised the paper; final approval of the version to be published. All authors have read and agreed to the published version of the manuscript.

## Funding statement

This work was supported by the Ethiopia Sheep and Goat Productivity Improvement Program (ESGPIP), ARARI (Amhara 10.13039/501100013249Agricultural Research Institute), 10.13039/501100004535EIAR (Ethiopian 10.13039/501100013249Agricultural Research Institute), 10.13039/501100007941Addis Ababa University 10.13039/100011523College of Veterinary Medicine and Agriculture (CVMA), and, International 10.13039/501100013249Agricultural Research Institute in Dry Areas (ICARDA).

## Data availability statement

All data generated or analyzed during this study can be available on request to the corresponding author.

## Ethics approval

The manuscript does not contain clinical studies or patient data.

## Additional information

No additional information is available for this paper.

## Declaration of AI and AI-assisted technologies in the writing process

Not applicable.

## Declaration of competing interest

The authors declare that they have no known competing financial interests or personal relationships that could have appeared to influence the work reported in this paper.
